# Changes in the Cell Wall Proteome of Leaves in Response to High Temperature Stress in *Brachypodium distachyon*

**DOI:** 10.3390/ijms22136750

**Published:** 2021-06-23

**Authors:** Artur Pinski, Alexander Betekhtin, Bozena Skupien-Rabian, Urszula Jankowska, Elisabeth Jamet, Robert Hasterok

**Affiliations:** 1 Plant Cytogenetics and Molecular Biology Group, Institute of Biology, Biotechnology and Environmental Protection, Faculty of Natural Sciences, University of Silesia in Katowice, 40-032 Katowice, Poland; robert.hasterok@us.edu.pl; 2 Proteomics and Mass Spectrometry Core Facility, Malopolska Centre of Biotechnology, Jagiellonian University, 31-007 Krakow, Poland; bozena.skupien-rabian@uj.edu.pl (B.S.-R.); urszula.jankowska@uj.edu.pl (U.J.); 3 Laboratoire de Recherche en Sciences Végétales, Université de Toulouse, CNRS, UPS, 31326 Auzeville Tolosane, France; jamet@lrsv.ups-tlse.fr

**Keywords:** *Brachypodium distachyon*, cell wall proteomics, glycoside hydrolase, leaves, high temperature stress

## Abstract

High temperature stress leads to complex changes to plant functionality, which affects, i.a., the cell wall structure and the cell wall protein composition. In this study, the qualitative and quantitative changes in the cell wall proteome of *Brachypodium distachyon* leaves in response to high (40 °C) temperature stress were characterised. Using a proteomic analysis, 1533 non-redundant proteins were identified from which 338 cell wall proteins were distinguished. At a high temperature, we identified 46 differentially abundant proteins, and of these, 4 were over-accumulated and 42 were under-accumulated. The most significant changes were observed in the proteins acting on the cell wall polysaccharides, specifically, 2 over- and 12 under-accumulated proteins. Based on the qualitative analysis, one cell wall protein was identified that was uniquely present at 40 °C but was absent in the control and 24 proteins that were present in the control but were absent at 40 °C. Overall, the changes in the cell wall proteome at 40 °C suggest a lower protease activity, lignification and an expansion of the cell wall. These results offer a new insight into the changes in the cell wall proteome in response to high temperature.

## 1. Introduction

In recent years, plant productivity has been increasingly threatened by climate change, primarily extreme climate events, altered rainfall patterns and increasing global mean land and ocean surface temperatures [1]. Multimethod analyses predict that each Celsius degree increase in the global mean temperature will negatively impact the yields of four major crops: maize, wheat, rice and soybean, which will result in food scarcity. However, it should be noted that the results are highly heterogeneous regarding geographical areas and crops with some estimations of a positive impact [2]. Simultaneously, there is also a need to increase food production because of the growing population (an additional two billion-plus people by 2050), a decrease of arable land and switch in the use of land for biofuel production and other industries [3]. In order to cope with the negative impact of climate changes and need to increase food production, there is an urgency to develop new climate-smart crops that fit into the climate-smart agriculture approach [3,4,5]. The development of such crops can be done using genetic engineering and breeding as well by exploiting epigenetic variations [6,7]. However, this requires detailed knowledge about the molecular mechanisms that underlie the various stress responses [8,9,10,11,12]. In order to facilitate the development of climate-smart crops, *Brachypodium distachyon* was proposed as a model plant for temperate grasses [13,14] because it offers a suite of advantages: undemanding growth requirements, small stature, short life cycle, relatively small genome and established protocols for efficient genomic editing [3,15,16,17,18]. Large collections of natural accessions with well-described genetic diversity and variation in DNA methylation are also available [19,20]. *B. distachyon* constitutes a useful target for genetically manipulating the cell wall composition as the cell wall of grasses differs from that of dicots in the aspects of major structural polysaccharides and the abundance of pectins, phenolic compounds and proteins [14,21]. The most notable and exciting area for discovery is that of lignin formation in grasses [16].

Temperature stress induced by high temperatures, leads to complex changes in the photosynthesis efficiency, reactive oxygen species scavenging, redox adjustment, cytoskeletal rearrangements and cell wall remodelling [22,23]. Although they constitute less than 10% of the dry mass for dicots and 1% for monocots, the cell wall proteins (CWPs) participate in cell wall remodelling, signalling and its overall scaffolding structure [9,14,24,25]. To facilitate their analysis, CWPs are usually divided into nine functional classes: proteins acting on carbohydrates, oxido-reductases, proteases, proteins related to lipid metabolism, proteins that are possibly involved in signalling, proteins that have predicted interaction domains, miscellaneous proteins, structural proteins and proteins with an unknown function. CWPs can be identified through a proteomic analysis using liquid chromatography tandem-mass spectrometry (LC-MS/MS). The process begins with the purification of the cell walls, followed by the extraction of CWPs using salt solutions. It is difficult to obtain a full representation of CWPs because the cell wall is an open compartment that has no surrounding structure, which results in the loss of some CWPs during the isolation procedure and because some CWPs are covalently linked to other cell wall components. Additionally, the isolated CWPs are contaminated with intracellular proteins that require further bioinformatic analysis [24,25]. Using such approaches, has enabled 699 *B. distachyon* CWPs to be identified in the leaves, stems and seeds (data available at *WallProtDB*, http://www.polebio.lrsv.ups-tlse.fr/WallProtDB/, accessed on 29 March 2021, [26]). 

In a previous work, we investigated the response of the hydroxyproline-rich glycoproteins (HRGPs) to temperature stress in leaves. We showed a differential response of the HRGPs to temperature stress with more pronounced changes in the levels of the mRNAs encoding the AGPs at a high temperature [27]. Although the roots and reproductive organs are more sensitive to temperature, the response of the leaves directly affects plant productivity [9,28].

However, it is known that high temperature stress affects other CWPs beside HRGPs [9] but there are only a few studies on monocots [29,30]. In order to analyse these changes in the cell wall proteome of *B. distachyon* more broadly, a cell wall proteomics approach was used. This study aimed to characterise the changes in the cell wall proteome of *B. distachyon* leaves in response to high (40 °C) temperature stress.

## 2. Results

### 2.1. Isolating the Cell Wall Proteins

**Figure A1:**
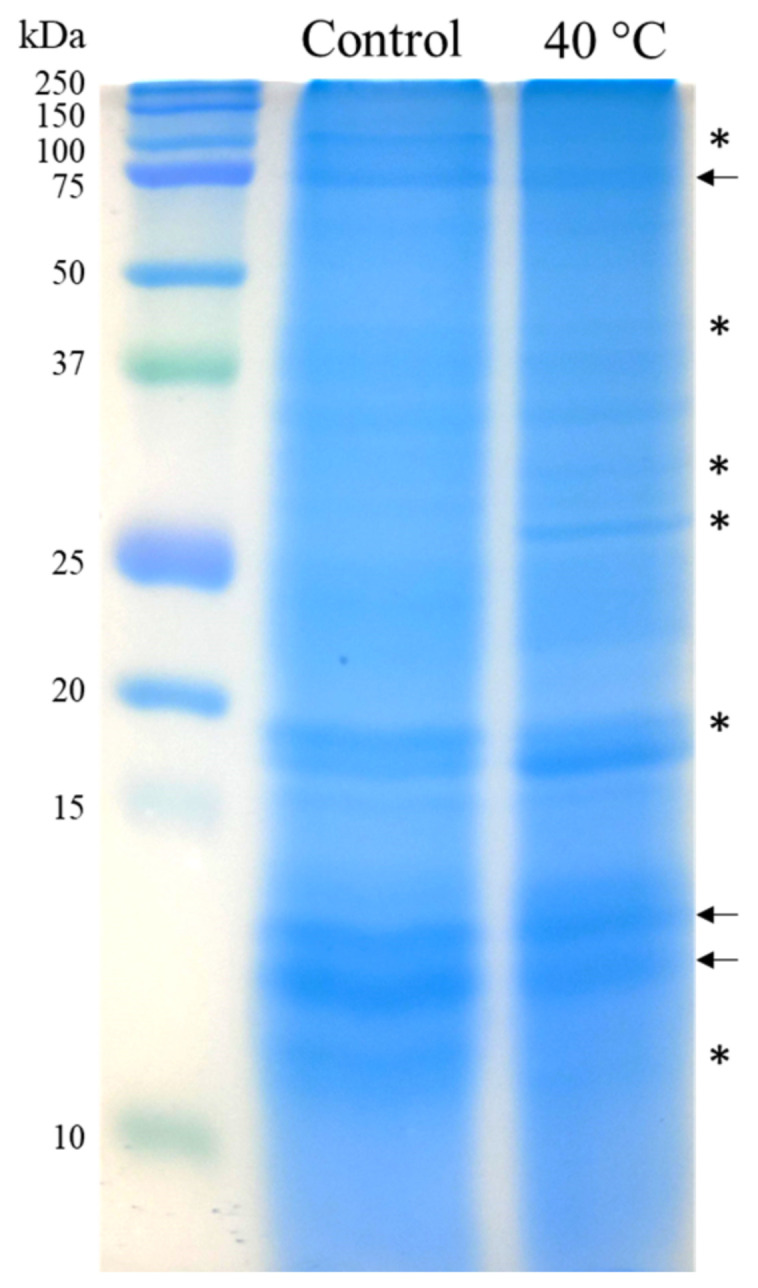
The 1D-electrophoretic patterns of the proteins that were isolated from the purified cell walls of the *B. distachyon* leaves that had been treated at 40 °C and the control. Sixty µg of the proteins were analysed for each sample. After electrophoresis, the proteins were stained with Coomassie blue. Some of the bands of similar intensity across all treatments are marked with arrows. The bands differentiating plants treated at 40 °C from those at the control are marked with asterisks.

The CWPs were isolated from about 3 g of the fresh leaves of *B. distachyon,* which enabled about 0.75 mg proteins (0.25 mg proteins per g fresh leaves) to be extracted. The proteins, which were separated using 1D-electrophoresis, showed a reproducible band pattern across all four of the biological replicates. None of the samples showed visible protein degradation patterns with presence of distinct bands from the largest proteins to the smallest ones. The 1D-electrophoretic patterns were slightly different between the control (21 °C) and plants subjected to 40 °C treatment (Figure A1).

### 2.2. Overall Proteome Data Analysis 

In the two different treatments (control and 40 °C), 1533 non-redundant proteins were identified through an LC-MS/MS analysis. Of these, only 338 CWPs were identified (22.05% of the total identified unique proteins) based on the prediction of a signal peptide by at least one bioinformatics tool (SignalP, TargetP or Phobius) and the absence of any known intracellular retention domain [26]. A protein was validated in a given sample when at least two unique peptides were present and in a given treatment when it was present in at least three of the four biological replicates. To make sure that high temperature treatment was not deleterious to plants, the amount of a few proteins related to the cell death process was checked, namely Bradi5g25970, Bradi1g37780, Bradi4g28680, Bradi1g60800, Bradi1g64680, Bradi2g02000, Bradi2g52470, and Bradi4g44667 [31,32]. No change in their level of accumulation was observed. 

**Figure 1:**
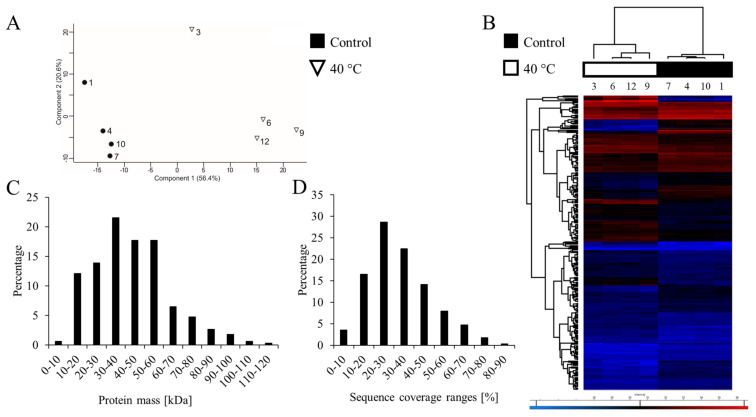
General features of the proteomic analysis. (**A**) Principal component analysis scatterplot in which each point represents a biological replicate. (**B**) The hierarchical clustering of the differentially abundant proteins (DAPs) that were identified in the experiment. (**C**) The molecular mass distribution of the proteins. The X-axis represents the theoretical molecular mass (kDa); the Y-axis represents the percentage of CWPs within each mass range. (**D**) Sequence coverage distribution for the identified proteins. The X-axis represents the sequence coverage ranges; the Y-axis represents the percentage of CWPs within each sequence coverage range.

In this study, 39 new CWPs were identified compared to those that were already recorded in *WallProtDB*, which increases the number of experimentally validated CWPs in *B. distachyon* to 738. The number of CWPs that were identified for each treatment differed, and the highest number in the control—311 (21.9%) and the lowest number at 40 °C -253 (16.8%) (Table S1). The number of identified CWPs was different between the control and 40 °C; fewer CWPs were present predominantly in the proteins acting on cell wall polysaccharides and oxido-reductases functional classes at 40 °C (Table 1). The principal component analysis showed that the samples from 40 °C were grouped together and were separate from the control, which suggests significant differences between them (Figure 1A). The first factor explained 56.4% of the changes between the control and samples at 40 °C. Moreover, a hierarchical clustering for CWPs showed the control samples with samples at 40 °C forming a separate branch (Figure 1B). The protein molecular masses were predominantly concentrated in the 20-60 kDa range, but proteins up to 120 kDa were also identified, which showed a good coverage of the different molecular masses (Figure 1C). In most of the proteins, there was high protein sequence coverage with the identified peptides (Figure 1D). A sequence coverage of more than 20% was observed for 80% of the proteins.

### 2.3. Qualitative Analysis

For the qualitative analysis, only CWPs were included. The percentage of the contributions of the functional classes was different for each treatment and the differences were most prominent in the classes of proteins acting on the cell wall polysaccharides, proteases, oxido-reductases and miscellaneous proteins (Table 1). A lower contribution was observed at 40 °C (15.4% vs. 16.7% in the control) for the proteins acting on cell wall polysaccharides and oxido-reductases. Simultaneously, there was a higher representation of the miscellaneous proteins at 40 °C (13.0%) than in the control (11.3%). Using a more rigorous approach, a protein was found to be exclusively present in a given treatment when it was identified in all four biological replicates and was absent in the other temperature treatment. This approach revealed 25 differentially present CWPs (Table S1). One protein was found to be exclusively present at 40 °C (Bradi4g14920) while being absent in the control and 24 proteins were found to be present in the control but not at 40 °C. 

### 2.4. Quantitative Analysis

Only CWPs were considered for the quantitative analysis. To identify the differentially abundant proteins (DAPs), a protein had to be present in at least three of the four biological replicates for a given treatment. Considering the differences between the control and 40 °C, 46 DAPs were identified. Of these, 4were over-accumulated and 42 were under-accumulated. A classification of CWPs into the functional classes showed changes in the abundance of CWPs in seven; both an over-accumulation and under-accumulation were observed in three of them (Table 2). Notably, the most significant changes were in the class of proteins acting on cell wall polysaccharides with 2 over- and 12 under-accumulated proteins. Looking at the level of protein families, most of the DAPs were classified as glycoside hydrolases (GHs) (11 DAPs), Asp proteases (6), class III peroxidases (CIII Prxs) (4) and GDSL-lipases/acylhydrolases (4) (Table S1). The abundance differences ranged from a 23.2-fold increase at 40 °C for Bradi4g09430 (xylanase inhibitor/class II chitinase of the GH18 family according to the CAZy database, http://www.cazy.org/, accessed on 29 March 2021) to a 15.8-fold decrease for Bradi1g52050 (polygalacturonase of the GH28 family according to the CAZy database) compared to the control (Table 3).

### 2.5. Expression of Six Genes Encoding the DAPs

**Figure 2:**
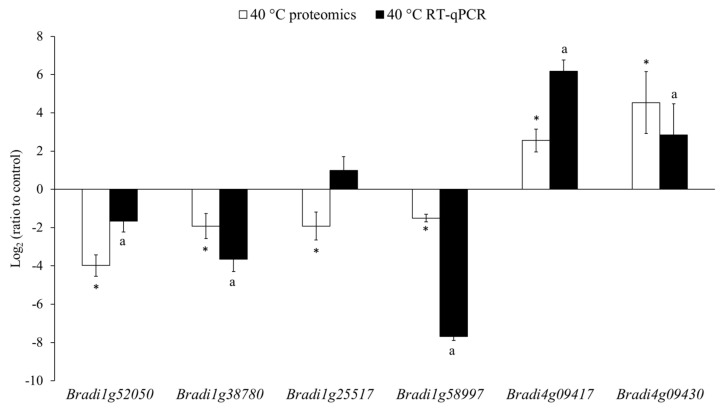
Comparison between the relative expression levels of the six genes that were measured using RT-qPCR and the relative level of the accumulation of the encoded proteins. *Bradi1g52050* encodes a polygalacturonase (GH28), *Bradi1g38780* a GDSL-lipase/acylhydrolase, *Bradi1g25517* an endo-β-1,3-glucosidase (GH17), *Bradi1g58997* a CIII Prx (BdiPrx35), *Bradi4g09417* a class II chitinase (GH18) and *Bradi4g09430* another GH18. The polyubiquitin gene (*Bradi1g32860*) was used as the reference gene for the RT-qPCR measurements and the control treatment (at 21 °C) as the reference group for all the calculations. The error bars of RT-qPCR results represent the standard deviations that were calculated from three biological replicates and two technical replicates for each biological replicate. The letter “a” indicates significant differences from the control using the Student’s t-test (*p* < 0.05; mean ± SD, *n* = 3). The error bars indicated for the quantitative proteomic results represent the standard deviations that were calculated from four biological replicates. Asterisks indicate significant differences from the control using the Student’s *t*-test with the permutation-based FDR set to 1% (q-value < 0.01).

RT-qPCR was used to analyse the expression levels of six genes encoding the DAPs and showed a contrasting differential level of accumulation between the 40 °C treatment and the control (Table 3): *Bradi1g52050* encodes a polygalacturonase (GH28), *Bradi1g38780* a GDSL-lipase/acylhydrolase, *Bradi1g25517* an endo-β-1,3-glucosidase (GH17), *Bradi1g58997* a CIII Prx (BdiPrx35), *Bradi4g09417* a class II chitinase (GH18) and *Bradi4g09430* another GH18. The relative expression levels of five genes at 40 °C showed similar trends as those for the proteomics results (Figure 2). In contrast, the relative expression levels of one remaining gene had a trend opposite to the result of the proteomic analysis, although the differences were small. It is worth noting that the proteomic analysis showed changes in the abundancy of proteins that were accumulated or degraded during the entire treatment period (24 h), while the expression analysis showed the levels of gene expression at a specific timepoint (after 24 h). Moreover, the relationship between the expression level of a given gene and the biosynthesis of the encoded protein is complex and is subject to various levels of regulation, which makes proteomic analysis more informative regarding the actual changes in the cell wall proteomes [33,34].

## 3. Discussion

### 3.1. Proteins Acting on Cell Wall Polysaccharides

Glycoside hydrolases (GHs) are enzymes that are able to rearrange the plant cell wall polysaccharides, thereby participating in plant development and the response to stress [35]. The treatment at 40 °C resulted in significant changes in the proteins that belong to the GH family with an over-accumulation of two GH18, an under-accumulation of 11 GHs (GH1, GH3, GH5, GH16, GH17, GH19 and GH28) and the absence of three GHs (GH1, GH3 and GH10). GH18 is among the most interesting GH families as its members can be xylanase inhibitors or class II chitinases. This family has more representatives in grasses than in dicots, thus they constitute promising candidates for further investigating their role in the cell wall [36]. The Bradi4g09430 protein, which was over-accumulated at 40 °C, was characterised as a xylanase inhibitor [37]. Such proteins reversibly inhibit the xylanases that belong to GH10 (the Bradi5g04640 GH10 was absent at 40 °C) and GH11 [38]. The inhibition of xylanases can limit the early stages of fungal infections [37]. GH18 can also work in synergy with GH19 (chitinases) to promote antifungal activity [39]. The members of GH3 family exhibit broad substrate specificities acting on arabinoxylans and (1-3)(1-4)-β-D glucans, which are the most common hemicelluloses in grasses [40]. GH16 have xyloglucan endotransglucosylase/hydrolases activity and are likely to be involved in xyloglucan remodelling, although their effect on the cell wall mechanics seems to be relatively weak [41]. GH1 exhibit a β-glycosidases activity and are involved in lignification, the defence against herbivory and the hydrolysis of the cell wall-derived oligosaccharides during germination [42]. Finally, GH28 exhibits polygalacturonase activity, which catalyses pectin depolymerisation, which can result in cell wall loosening [39]. 

Previous studies have indicated the role of expansins in the heat stress response through the loosening of the cell walls [9]. In our experiment, we identified three expansins that were present in the control but were absent at 40 °C. Such an absence aligns with previous experiments, which showed differential changes in the expression of the expansin genes that are specific to a species and/or the expansin isoforms [9]. For example, in *Brassica napus* seedlings, the expression of *EXPA5* was reduced ten-fold at 38 °C, and in the case of *Populus simonii*, 13 genes encoding expansins were also down-regulated at 42 °C [43,44]. On the other hand, in *Agrostis scabra,* the up-regulation of one of expansin gene was positively correlated with a better heat tolerance [45]. Indeed, a few studies have suggested that although the cell wall loosening by expansins promotes plant growth and development, it makes plants more vulnerable to biotic and abiotic stresses [46,47]. 

The trichome birefringence-like (TBL) proteins through the O-acetylation of cell wall polysaccharides (either hemicelluloses or pectins) affect their physicochemical properties of polymers [48]. Notably, as has been shown in *Arabidopsis thaliana,* the TBL proteins are essential for the plant response to various biotic and abiotic stresses [49]. In *A. thaliana,* exposure to aluminium resulted in a decrease in the *TBL27* transcript accumulation and the mutant in this gene had a higher aluminium accumulation in the hemicellulose fraction than the wild type [48]. In *Oryza sativa*, a double mutant that was impaired in the TBL genes (*tbl1 tbl2*) exhibited a stunted growth phenotype with varying degrees of dwarfism [50]. In response to heat stress, we observed a decreased abundance of the Bradi2g45230 TBL protein and the lack of another one (Bradi2g07000), which were both present in the control. This could lead to a lower level of the O-acetylation of the cell wall polymers.

Pectins can undergo various modifications in the cell wall, among which is the demethylesterification by pectin methylesterases (PMEs) [51]. One PME was absent at 40 °C compared to the control together with one pectate lyase that catalyses the eliminative cleavage of de-esterified pectin. The changes in the degree of pectin methylesterification and its consequence of the formation of the so-called egg-boxes with the calcium ions have previously been described, although the mechanism and effect on heat tolerance remain to be elucidated [52,53].

### 3.2. Proteases

The proteases break the peptide bonds and control key plant processes such as protein transport, activity and half-live [36]. Our results revealed the under-accumulation of several proteases (aspartyl, cysteine and serine) and the absence of a few proteases (aspartyl, cysteine, serine and peptidase C13) at 40 °C, although they were present in the control. Only one peptidase that belongs to the M20 family was over-accumulated at 40 °C. Overall, at 40 °C, the presence of the proteases seemed to decrease compared to the control, which suggests a lower proteolytic activity. The *Bradi5g26620* and *Bradi5g16960* encoding proteases, which were absent at 40 °C, have been proposed as novel candidate genes that might participate in grain programmed cell death [54].

### 3.3. Miscellaneous Proteins and Proteins of a Yet Unknown Function

The miscellaneous class includes CWPs with diverse functions, among others, the dirigent proteins, which are involved in lignan and lignin biosynthesis that are involved in plant development and the response to a pathogen infection or abiotic stress [36,55]. In our analysis, one over-accumulated and one under-accumulated dirigent protein were identified. Numerous genes encoding the dirigent proteins were found to be up-regulated in response to salt stress in the roots of *B. distachyon,* among which *Bradi1g20950* encoding a CWP that was found among the over-accumulated CWPs in this experiment [56]. In *Medicago sativa*, a gene encoding a dirigent protein was up-regulated due to heat stress [57]. Though a few studies have shown changes in the expression of the genes encoding the dirigent proteins, the molecular mechanisms underpinning their involvement in stress responses remain to be elucidated [55]. The next group of miscellaneous proteins - the germin proteins - has also been indicated to respond to abiotic stresses with genes that exhibit various expression changes as has been shown for rice that had been subjected to desiccation, salt or cold stress [58]. The one germin protein was under-accumulated at 40 °C compared to the control. Another protein, an SCP-like extracellular protein, was also found to be under-accumulated. Dienelactone hydrolase catalyses the conversion of dienelactone to maleylacetate and participates in the degradation of the haloaromatic compounds. The under-accumulation of a dienelactone hydrolase (Bradi3g18680) that was observed in this study may cause a decrease in the degradation of these secondary metabolites as has been suggested to explain a similar decrease in the abundance of a dienelactone hydrolase in the roots of soybean in response to a NaCl treatment [59]. Although the DUF642 protein family has been shown to be involved in the development and growth of plants and the response to various stresses, its precise function remains unknown [60]. In the fruit mesocarp of *Prunus persica*, the under-accumulation of a DUF642 protein was observed after a post-harvest heat treatment [61]. There was a similar under-accumulation of Bradi2g43230, which belongs to the DUF642 protein family. At the same time, a DUF538 protein was identified in the control, while it was absent at 40 °C. The DUF538 proteins are predicted to have esterase-type hydrolytic activity towards the bacterial lipopolysaccharides and chlorophyll molecules. Recently, the DUF538 proteins were also predicted to have pectin methylesterase activity [62].

### 3.4. Oxido-Reductases

The CIII Prxs belong to a large multigenic family and have dual hydroxylic and peroxidative cycles that allow them to build rigid cell walls or loosen them [63]. The specific function of a given CIII Prx remains elusive due to their substrate diversity and spatiotemporal regulation of the expression of their genes. However, for some CIII Prxs of *A. thaliana*, a role in cell wall dynamics could be attributed [63]. In the *B. distachyon* genome, 143 CIII Prxs genes have been annotated, though none of them was linked with a specific function [63,64]. In our experiment, we found four under-accumulated CIII Prxs at 40 °C and one only present in the control. We also observed the under-accumulation of a laccase and a blue copper-binding protein and the absence of one multicopper oxidase and a blue copper-binding protein at 40 °C. Taken together, this suggests a perturbation in the oxidative state of the cell wall and decreased lignification processes under heat stress.

### 3.5. Proteins Related to Lipid Metabolism

The GDSL-lipases/acylhydrolases play essential roles in plant growth and development, organ morphogenesis, the stress response and lipid metabolism as well as hydrolysing and synthesising various lipids [65]. Though genes encoding GDSL-lipases/acylhydrolases are usually up-regulated in response to biotic stresses, a few experiments have also suggested their role in abiotic stresses. For example, an overexpression of *A. thaliana LTL1* leads to an increased salt tolerance [66]. Notably, the expression of GDSL-lipases/acylhydrolases varies significantly in response to salt stress as was shown for *B. distachyon* roots [56]. In the experiment presented here, four GDSL-lipases/acylhydrolases were under-accumulated at 40 °C, suggesting a decrease in hydrolysis and lipid synthesis in response to heat stress. Additionally, two GDSL-lipases/acylhydrolases present in the control were absent at 40 °C (Bradi3g39622 and Bradi1g01920). The under-accumulation of a glycerophosphoryl diester phosphodiesterase (GDPL) could suggest some changes in the primary cell wall organisation since it was shown that a mutant of *A. thaliana* with inactivated *SHV3* encoding a GDPL exhibited a ruptured root hair phenotype. This phenotype was suppressed by adding borate, which is involved in rhamnogalactunonan II cross-linking [67].

### 3.6. Proteins with Interaction Domains (with Proteins or Polysaccharides)

At 40 °C, the under-accumulated Bradi4g37090 protein had two LysM domains with a possible glycosylphosphatidylinositol anchor. Though proteins with the LysM domain can have different structures, they mediate the recognition of the different N-acetylglucosamine-containing ligands that facilitate microbial infection and symbiosis [68]. In our data, we also found a protein with a Barwin domain (Bradi4g14920) that was only present at 40 °C. The Barwin domain itself can bind carbohydrates and has an antifungal, ribonuclease or DNase activity [69]. Interestingly, proteins with this domain were previously observed to be over-accumulated in response to heat stress (after 6 h and 12 h) in rice [70]. The gene encoding this protein with a Barwin domain was also found to be upregulated in *B. distachyon* roots inoculated with *Pseudomonas fluorescens* SBW25 [71]. Taken together, this suggests the importance of Bradi4g14920 in the response to heat stress as well as in root defence mechanisms in response to bacterial colonisation. Such involvement in different stresses can be explained by the crosstalk between cellular signalling events [72]. Though, recent studies have shown that the plant responses to combinations of multiple stress conditions are unique and require species-specific studies [73].

### 3.7. Cell Wall Response to Temperature Stress

The changes in the cell wall proteome of the leaves at 40 °C compared to the control suggest a lower protease activity, lignification, an expansion of the cell wall and changes in the architecture of the cell wall polymers, especially the pectins. This enriches our understanding of the heat stress response that was robustly summarised by Le Gall et al. [9], where the authors highlighted the changes in the cell wall polysaccharides, cellulose, hemicelluloses and pectins, as well as lignin biosynthesis in response to heat stress. Moreover, *AGPs* are usually up-regulated under heat stress, which we showed in our previous work using RT-qPCR, and specific AGP epitopes accumulate as was shown by immunochemistry using specific antibodies. Interestingly, we could not identify any AGPs among the DAPs, which could be related to the fact that the changes in the AGP composition that were revealed by immunohistochemistry were tissue specific. However, one FLA (Bradi1g76630) was absent at 40 °C but was present in the control. The overall increase in their abundance might not be high enough to be significantly different between the treatments [27]. Alternatively, because these proteins are difficult to detect due to their small size and high level of glycosylation, they require specific approaches that begin with a deglycosylation step [74]. Thus, AGPs may have escaped our analysis like EXTs that are usually covalently cross-linked to other cell wall components [75]. Our study complements previous transcriptomic analysis of *B. distachyon* leaf response to the heat stress. In *B. distachyon* plants that have been treated at 42°C during 5 h, Chen et al. [29] observed differences in the expression of genes related to the photosynthesis-antenna proteins and the endoplasmic reticulum. Notably, high temperature also induced alternative splicing by up-regulation of gene expression involved in RNA splicing.

## 4. Materials and Methods

### 4.1. Plant Material and Treatment

Plants of the *B. distachyon* reference genotype Bd21 (accession number: PI 254867) were used and were treated as was previously described [27]. Briefly, the plants were grown in a greenhouse under a 16 h/8 h light/dark photoperiod at 21 ± 1 °C for four weeks and were illuminated by lamps emitting white light at an intensity of 150 μmol photons m^-2^ s^-1^. The plants were then transferred to growth chambers for 24 h at 40 °C for the high temperature treatment. For the control treatment, the plants were kept at 21 °C. The relative humidity was maintained at around 50% for all temperatures. For each of four biological replications of the control and 40 °C treatment, plants from subsequent cultivations were used.

### 4.2. Cell Wall Proteins Isolation

To isolate CWPs, whole leaves of *B. distachyon* (from the control and 40 °C treatment) were collected. For each experiment, around 3 g fresh leaves were used. The cell wall was extracted as was previously described [76,77]. The samples were washed in distilled water and placed in a blender with 100 mL of a 5 mM acetate buffer (Merck, Darmstadt, Germany) (pH 4.6) that had been supplemented with 0.4 M sucrose (Chempur, Piekary Slaskie, Poland), a protease inhibitor cocktail (55 µL/g material) (P9599, Merck) and 0.5 g polyvinylpyrrolidone (Merck). The mixture was ground in the blender at full speed three times for 2 min. The cell walls were separated from the cytoplasmic fluids by centrifugations (Eppendorf AG, Hamburg, Germany) for 15 min at 3000 g at 4 °C. The supernatant was gently decanted, and the pellets were washed with a cold 5 mM acetate buffer (pH 4.6) that had been supplemented with 0.6 M sucrose and centrifuged again for 15 min at 3000 g at 4 °C. The supernatant was gently decanted, and the cell walls were washed with cold 5 mM acetate buffer (pH 4.6) that had been supplemented with 1 M sucrose. Lastly, the samples were centrifuged for 15 min at 3000 g at 4 °C. After the supernatant was removed, the pelleted material was placed on a nylon net (25 µm pore size) and washed with 1 L of a 5 mM acetate buffer (pH 4.6) to remove the sucrose. Then, the material was thinly spread in a plastic bag and frozen at −80 °C. The frozen material was ground in liquid nitrogen in a mortar with a pestle and lyophilised (Christ, Osterode am Harz, Germany) for two days. The dried powder was used to extract CWPs using salt solutions. Five mL of 0.2 M CaCl_2_ in a 5 mM acetate buffer (pH 4.6) that had been supplemented with a 3.75 µL protease inhibitor cocktail was added to each sample. The samples were then vortexed for 30 min at 4 °C and centrifuged for 15 min at 5000 g at 4 °C. The supernatant was collected in a fresh tube and the extraction was repeated once with 0.2 M CaCl_2_ (Merck) in a 5 mM acetate buffer (pH 4.6) and twice with 2 M LiCl (Merck) in a 5 mM acetate buffer (pH 4.6). The collected supernatants were desalted using Econo-Pac 10 DG columns (Bio-Rad, Hercules, CA, USA) that had been equilibrated with 0.1 M formic acid ammonium salt (Merck). The desalted samples were lyophilised and resuspended in distilled water. The protein concentration was measured using the Pierce BCA Protein Assay Kit according to the manufacturer’s instructions (Thermo Fisher Scientific, Waltham, MA, USA). For each sample, 200 µg of the isolated proteins were transferred into separate tubes, lyophilised and saved for further proteomic analysis.

### 4.3. Sodium Dodecyl Sulphate Polyacrylamide Gel Electrophoresis (SDS-PAGE)

The quality and integrity of the isolated proteins were checked by separating them using SDS-PAGE. For the SDS-PAGE, a 12% separating gel was prepared, and for each well, 60 µg of the protein sample was mixed with a 4× Laemmli Sample Buffer (Merck) and heated in a water bath for 10 min before being applied to the gel [78]. As a protein ruler, a 5 µL Precision Plus Protein Kaleidoscope Prestained Protein Standards (Bio-Rad), 10 to 250 kDa, was used. After electrophoresis, the gel was washed three times with distilled water for 15 min. Then, it was stained with Coomassie^®^ blue using an EZBlue^TM^ Gel Staining Reagent (Merck) for 1 h and then washed three times with distilled water for 15 min and then overnight. The gels were scanned using a Brother MFC-J3530DW Business Smart Inkjet Printer All-in-One (Brother Industries, Nagoya, Japan).

### 4.4. Protein Tryptic Digestion and LC-MS/MS Analysis

The isolated proteins were suspended in a urea solution (8 M urea in 50 mM ammonium bicarbonate; BioShop Canada Inc., Burlington, Canada and Merck, respectively) to a concentration of 2 µg/µL and then 100 µg of proteins were prepared according to the FASP protocol [79]. The protein samples (100 µg) were diluted four times with the urea solution, reduced with 50 mM dithiothreitol (BioShop Canada Inc.) and incubated for 15 min at room temperature. Then, the samples were centrifuged (21 000 g, 15 min, 20 °C), transferred into filtration columns (30 kDa membrane cut-off, VIVACON 500, Sartorius Stedim Biotech GmbH, Göttingen, Germany) and centrifuged (14 000 g, 15 min, 20 °C). The 30 kDa membrane cut-off was used to increase the peptide yield and decrease sample preparation time while retaining small proteins [80]. The proteins remaining on the filter were washed with 200 µL of the urea solution and alkylated with 54 mM iodoacetamide (Merck) (20 min of incubation at room temperature in the dark). In the following steps, the proteins were washed three times with 100 µL of the urea solution and then three times with 100 µL of 50 mM ammonium bicarbonate (ABC) (Merck). After washing, trypsin (Promega, Madison, WI, USA) was added to the samples with an enzyme to protein ratio of 1:60. The lyophilised enzyme was suspended in 50 mM ABC to a final volume of 75 µL per sample. Digestion was performed overnight at 37 °C. The next day, the columns were transferred into new collection tubes, 40 µL of 50 mM ABC were added to the columns and the samples were centrifuged (14 000 g, 10 min, 20 °C). Then, the peptides were eluted with 40 µL of 50 mM ABC and 50 µL of 0.5 M NaCl. The obtained filtrate was collected and vacuum dried. The resulting peptide mixture was suspended in 100 µL of a loading buffer (2% acetonitrile with 0.05% trifluoroacetic acid; JT Baker, Phillipsburg, NJ, USA and Merk, respectively) and 1 µL of the solution was used for the LC-MS/MS analysis, which was performed on a Q Exactive mass spectrometer (Thermo Fisher Scientific) equipped with a PicoView nanospray source (DPV-550, New Objective, Aarle-Rixtel, The Netherlands) and coupled with a nanoHPLC (UltiMate 3000 RSLCnano System, Thermo Fisher Scientific). The peptides were loaded onto a pre-column (AcclaimPep-Map100 C18, Thermo Fisher Scientific; ID 75 μm, length 20 mm, particle size 3 μm, pore size 100 Å) and then eluted from an analytical column (AcclaimPepMapRLSC C18, Thermo Fisher Scientific; ID 75 μm, length 500 mm, particle size 2 μm, pore size 100 Å) in the presence of 0.05% formic acid (JT Baker) using a linear gradient of acetonitrile (2%–40%) for 240 min. The mass spectrometric measurement was performed in the data-dependent mode using the Top12 method. The MS and MS/MS spectra were acquired at resolutions of 70 000 and 17 500, respectively. The performance of the LC-MS/MS platform was monitored using the QCloud quality control system [81].

### 4.5. Bioinformatic Analysis 

The LC-MS/MS data were processed using the MaxQuant software package (version 1.5.8.3) including the Andromeda search engine [82,83]. The protein sequences of *B. distachyon* Bd21 were downloaded from the Phytozome database (https://phytozome.jgi.doe.gov/pz/portal.html, accessed on 10 February 2021) (version 3.2, number of sequences 56,847). The search was done using the default parameters with minor modifications. The standard list of variable modifications, methionine oxidation and the acetylation of the protein N-termini was completed with proline oxidation. The false discovery rate (FDR) for the peptide and protein identification was set to 1%. Label free quantification (LFQ) was also enabled. The resulting table with the protein group identifications and quantitative information (LFQ intensities) was uploaded to the Perseus platform for further processing and statistical analysis [84]. The protein groups from the decoy database, contaminants and proteins only identified by site were filtered out. The data were log-transformed, and the matrix was filtered for the protein groups that had valid LFQ values in at least three replicates within each group. Then, any missing values were imputed from a normal distribution. Principal component analysis and hierarchical clustering were performed in Perseus using the default settings. The Student’s t-test with the permutation-based FDR set to 1% was used to reveal changes between the treatments. The resulting list of differential protein groups was additionally filtered for the protein groups that had been identified based on at least two specific peptides. A fold change threshold of 1.5 was used. Moreover, the protein groups that had been detected in all of the replicates from one experimental group and none of the other group replicates were considered to be qualitative differences.

### 4.6. RT-qPCR Analysis

To correlate the proteomic analysis results, six genes were selected to characterise the level of their transcript accumulation using RT-qPCR. Three genes (*Bradi1g52050*, *Bradi4g09417* and *Bradi4g09430*) were selected based on the highest differences in the accumulation of the proteins between the control and the 40 °C treatment, and the three other genes (*Bradi1g38780*, *Bradi1g25517* and *Bradi1g58997*) were selected to represent proteins from different families. The RNA for the control and treated leaves were isolated as described previously [27]. The primers that were used are listed in Table A1. The detailed procedure for RT-qPCR was also previously described [27]. Since we have already shown that the level of expression of the polyubiquitin gene (*Bradi1g32860*) was stable under different temperature treatments it was used as a reference [27]. The analysis was performed using the 2^−∆∆CT^ method. The control treatment (at 21 °C) was used as the reference group. Three biological replicates were performed with two technical replicates for each. The analysis of the statistical differences between the treatments and the control was done with Student’s t-test (TIBCO Software Inc., Palo Alto, CA, USA). As mentioned in Section 4.4, the proteomics data were log-transformed, and the Student’s t-test with the permutation-based FDR set to 1% was used for statistical analysis of differences between the treatments and the control (Perseus, Max-Planck Institute of Biochemistry, Martinsried, Germany). 

## 5. Conclusions

In this work, we focused on the changes in the cell wall proteome in response to high (40 °C) temperature treatment. We showed that a high temperature resulted in qualitative and quantitative changes compared to the control (21 °C). The qualitative analysis indicated a lower contribution of the proteins acting on cell wall polysaccharides and oxido-reductases and a higher representation of the miscellaneous proteins at 40 °C. Further, a quantitative analysis revealed 46 DAPs: 4 were over-accumulated and 42 were under-accumulated. Overall, this suggests a lower protease activity, lignification, an expansion of the cell wall and changes in the architecture of the cell wall polymers, especially the pectins at 40 °C. Our results provide a new insight into the changes in the cell wall proteome in response to high temperature, and also present potential new targets for further studies of the cell wall response to high temperature stress, such as *Bradi4g09430* (GH18, xylanase inhibitor/class II chitinase) and *Bradi1g52050* (GH28, polygalacturonase).

## Figures and Tables

**Table 1:** Number of unique proteins and CWPs that were identified for the control and 40 °C and classified into the functional classes (classified as in *WallProtDB*). The percentage of the proteins identified in the functional classes compared to the total number of CWPs for each treatment is indicated in brackets.

Functional Class	Control	40 °C
Proteins acting on cell wall polysaccharides	87 (28%)	65 (25.7%)
Proteases	57 (18.3%)	48 (19%)
Oxido-reductases	52 (16.7%)	39 (15.4%)
Proteins related to lipid metabolism	38 (12.2%)	32 (12.6%)
Proteins with interaction domains (with proteins or polysaccharides)	9 (2.9%)	9 (3.6%)
Proteins possibly involved in signalling	7 (2.3%)	5 (2%)
Structural proteins	3 (1%)	3 (1.2%)
Miscellaneous	35 (11.3%)	33 (13%)
Unknown function	23 (7.4%)	19 (7.5%)
Total number of CWPs	311	253
Total number of unique proteins	1423	1505

**Table 2:** DAPs for the 40 °C treatment compared to the control and classified into their functional classes (according to the classification introduced in *WallProtDB*).

Functional Class	Over-Accumulated DAPs	Under-Accumulated DAPs
Proteins acting on cell wall polysaccharides	2	12
Proteases	1	11
Miscellaneous	1	4
Unknown function	-	2
Oxido-reductases	-	6
Proteins related to lipid metabolism	-	6
Proteins with interaction domains (with proteins or polysaccharides)	-	1
Total number	4	42

**Table 3:** DAPs for the 40 °C treatment compared to the control.

Protein	Fold Change	q-Value	Functional Class	(Putative) Function
Over-Accumulated DAPs at 40 °C				
*Bradi1g20950*	1.8	0.00518	Miscellaneous	dirigent protein
*Bradi4g02700*	2.9	0	Proteases	peptidase M20
*Bradi4g09430*	23.2	0.00190	Proteins acting on cell wall polysaccharides	GH18 (xylanase inhibitor/class II chitinase)
*Bradi4g09417*	5.9	0	Proteins acting on cell wall polysaccharides	GH18 (xylanase inhibitor/class II chitinase)
**Under-Accumulated DAPs at 40 °C**				
*Bradi3g18680*	−7.9	0.00229	Miscellaneous	dienelactone hydrolase
*Bradi4g41300*	−3.3	0.00285	Miscellaneous	dirigent protein
*Bradi3g37670*	−1.7	0.00407	Miscellaneous	germin (cupin domain)
*Bradi1g57590*	−5.6	0.00405	Miscellaneous	SCP-like extracellular protein (PR-1)
*Bradi3g59210*	−5.6	0.00133	Oxido-reductases	laccase
*Bradi4g05980*	−2.7	0.00139	Oxido-reductases	CIII Prx (BdiPrx117)
*Bradi1g58997*	−2.8	0	Oxido-reductases	CIII Prx (BdiPrx35)
*Bradi2g12170*	−2.5	0.00115	Oxido-reductases	CIII Prx (BdiPrx62)
*Bradi3g09080*	−1.7	0.00409	Oxido-reductases	CIII Prx (BdiPrx96)
*Bradi1g77560*	−2.4	0.00326	Oxido-reductases	plastocyanin (blue copper-binding protein)
*Bradi1g36160*	−1.7	0.00876	Proteases	Asp protease (Peptidase family A1)
*Bradi3g61060*	−2.6	0.00393	Proteases	Asp protease (Peptidase family A1)
*Bradi4g12160*	−2.7	0.00282	Proteases	Asp protease (Peptidase family A1)
*Bradi1g19070*	−3.1	0	Proteases	Asp protease (Peptidase family A1)
*Bradi2g25850*	−3.8	0.00567	Proteases	Asp protease (Peptidase family A1)
*Bradi3g56660*	−8	0	Proteases	Asp protease (Peptidase family A1)
*Bradi1g09729*	−1.9	0.00348	Proteases	Cys protease (papain family) (Peptidase family C1A)
*Bradi1g09737*	−2.2	0.00266	Proteases	Cys protease (papain family) (Peptidase family C1A)
*Bradi3g01320*	−1.5	0.00198	Proteases	Ser carboxypeptidase (Peptidase family S10)
*Bradi1g60600*	−1.7	0.00998	Proteases	Ser carboxypeptidase (Peptidase family S10)
*Bradi3g57140*	−3.1	0.00123	Proteases	Ser protease (subtilisin) (Peptidase family S8)
*Bradi2g45320*	−4.2	0.00155	Proteins acting on cell wall polysaccharides	expressed protein (Trichome Birefringence-like domain)
*Bradi1g10940*	−3.9	0.00301	Proteins acting on cell wall polysaccharides	GH1 (β-glucosidase)
*Bradi1g10930*	−4.3	0.00165	Proteins acting on cell wall polysaccharides	GH1 (β-glucosidase)
*Bradi3g18590*	−5.5	0	Proteins acting on cell wall polysaccharides	GH16 (endoxyloglucan transferase)
*Bradi1g25517*	−3.8	0.00279	Proteins acting on cell wall polysaccharides	GH17 (β-1,3-glucosidase)
*Bradi2g60441*	−5.8	0.00131	Proteins acting on cell wall polysaccharides	GH17 (β-1,3-glucosidase)
*Bradi2g47210*	−1.9	0	Proteins acting on cell wall polysaccharides	GH19
*Bradi1g52050*	−15.8	0	Proteins acting on cell wall polysaccharides	GH28 (polygalacturonase)
*Bradi1g08570*	−2	0.00902	Proteins acting on cell wall polysaccharides	GH3
*Bradi1g08550*	−7.1	0	Proteins acting on cell wall polysaccharides	GH3
*Bradi1g67760*	−1.6	0.00346	Proteins acting on cell wall polysaccharides	GH35 (β-galactosidase)
*Bradi2g31690*	−2.5	0.00116	Proteins acting on cell wall polysaccharides	GH5 (cellulase)
*Bradi1g14983*	−1.5	0.00398	Proteins related to lipid metabolism	glycerophosphoryl diester phosphodiesterase
*Bradi1g49010*	−2.4	0.00149	Proteins related to lipid metabolism	GDSL-lipase/acylhydrolase
*Bradi1g23120*	−3.5	0.00962	Proteins related to lipid metabolism	GDSL-lipase/acylhydrolase
*Bradi1g38780*	−3.8	0.00161	Proteins related to lipid metabolism	GDSL-lipase/acylhydrolase
*Bradi1g50897*	−7.4	0.00117	Proteins related to lipid metabolism	GDSL-lipase/acylhydrolase
*Bradi1g21870*	−6.3	0	Proteins related to lipid metabolism	lipid transfer protein/trypsin-alpha amylase inhibitor
*Bradi4g37090*	−3.5	0.00113	Proteins with interaction domains (with proteins or polysaccharides)	expressed protein (LysM domain)
*Bradi2g43230*	−2.2	0.00728	Unknown function	expressed protein (DUF642)
*Bradi3g29710*	−1.6	0.00943	Unknown function	Plant Basic Secreted Protein (BSP)

## Data Availability

The mass spectrometry data were deposited at the ProteomeXchange Consortium [85] via the MassIVE repository with the dataset identifier PXD025679. The cell wall proteomics data have been deposited at *WallProtDB* http://www.polebio.lrsv.ups-tlse.fr/WallProtDB/, accessed on 29 March 2021).

## Supplementary Materials

The following are available online at https://www.mdpi.com/article/10.3390/ijms22136750/s1.

## Appendix A

**Table A1:** The oligonucleotide primers that were used for the RT-qPCR analysis with the relevant descriptions of the genes.

Genes	Description of the Genes	Primer Sequences (5′→3′)
*Bradi1g32860*	Ubiquitin	GAGGGTGGACTCCTTTTGGA
		TCCACACTCCACTTGGTGCT
*Bradi1g52050*	GH28	CGACATGGTGGTCGAAATAC
		GTGACGTTGGAGATGAAGATG
*Bradi1g38780*	GDSL/lipase acylhydrolase	CCCTCTGTAAATCGGAGAAAG
		CGGAGAACAATGGAGCATTA
*Bradi1g25517*	GH17	GCGAGTTCAAAGACGACAT
		GTACGTGTAGTACGGGTAGAT
*Bradi1g58997*	class III peroxidase (BdiPrx35)	GGTCCTTCGACAACCAGTA
		TAGTCCTCGAGTCGGTGTA
*Bradi4g09417*	GH18	CTCCCTCATAGCTCTCCTATC
		GAGCCTTCGTCCTTGTTC
*Bradi4g09430*	GH18	AGGTTCTACATCGGGCTTAC
		GCCGTAGTTCTCCTTCTTCT
